# Static and Fatigue Strength and Failure Mechanisms of Riveted Lap Joints of CFRP Composites

**DOI:** 10.3390/ma16051768

**Published:** 2023-02-21

**Authors:** Jan Godzimirski, Marek Rośkowicz, Michał Jasztal, Iga Barca

**Affiliations:** Faculty of Mechatronics, Armament and Aerospace, Military University of Technology, 00-908 Warsaw, Poland

**Keywords:** riveted and hybrid joint, static strength, fatigue life, failure mechanism

## Abstract

The background of this work is the search for the most effective ways of joining composites, inter alia in aeronautical applications. The purpose of this study was to analyze the impact of mechanical fastener types on the static strength of lap joints of composite elements and the impact of fasteners on the mechanism of failure of such joints under fatigue load. The second objective was to check to what extent the hybridization of such joints, consisting of supplementing them with an adhesive joint, affects their strength and the mechanism of failure of such joints loaded with fatigue. Damage to composite joints was observed using computed tomography technology. The fasteners used in this study (aluminum rivets, Hi-lok and Jo-Bolt) differed not only in terms of the materials they were made of, but also in terms of the pressure forces they exerted on the joined parts. Finally, in order to check how a partially cracked adhesive joint affects the load on the fasteners, numerical calculations were carried out. Analyzing the results of the research, it was found that partial damage to the adhesive joint of the hybrid joint does not increase the load on the rivets and does not impair the fatigue life of the joint. An important advantage of hybrid joint is the two-stage destruction of the connection, which significantly increases the safety of aircraft structures and facilitates the process of supervising their technical condition.

## 1. Introduction

In recent years, there has been an increase in the use of composite materials (such as CFRP–Carbon Fiber Reinforced Polymer) in mechanical engineering, including the designs of currently manufactured aircraft. This is because of the significant advantages of composite materials, including but not limited to their lower density compared to aluminum alloys, which had dominated aircraft design for years. A valuable advantage of composite materials is also their very high fatigue life [1,2,3,4,5,6].

The problem, which is associated with the use of composite materials (e.g., CFRP) in mechanical engineering, is their lower resistance to surface pressures compared to structural metals [7,8,9], which is the reason why the search for the most effective ways to assemble components made of composites is still underway. Composite parts are joined by means of adhesive bonding, mechanical bonding, or by using both technologies in hybrid (adhesive–mechanical) joints. Mechanical or hybrid joints are recommended technologies for joining composite elements in aeronautical structures, due to the much higher diagnostic susceptibility of this type of joints, significantly higher fatigue life compared to, for example, adhesive bonds, easier certification process of this type of solution in aeronautical designs and, in the case of mechanical joints, an option of disassembling the fasteners [10,11,12].

In the design of mechanical joints, the geometry of fastener placement in the structural node is an important assembly factor. When joining composite parts, assembly recommendations related to the geometry of fastener placement (mounting hole design scheme) are even more stringent than for metal parts, due to the limited surface pressure resistance of the composite material [13]. Damage to composites resulting from exceeding their surface pressure strength comes in the form of the buckling of fibers “supported” by the impregnating agent at the level of the composite layer, the shearing of the adhesive at the fiber interface, and destruction of fibers released from the matrix [14,15,16].

The assembly problems of composite parts are further exacerbated by the layered structure of composite materials and the anisotropic properties of the composite material [17]. Therefore, in the case of composite materials, the execution of mounting holes itself becomes an important research issue and numerous studies have shown that the strength of mechanical joints depends on many factors, including, but not limited to, damage caused by the hole drilling process or excessive mounting clearances (hole and fastener fit) [18,19,20,21]. Furthermore, we can find work in the literature [22,23] about the effect of the layer number, orientation, material of the fiber-reinforced polymer, and joint geometry on the stress concentration factors in tubular X-connections retrofitted with FRP. In these papers, the initial stiffness, the ultimate capacity, the capacity ratio, and the failure mechanisms of the tubular X-joints reinforced with fiber-reinforced polymer (FRP) under compressive load are investigated with the use of a finite element model verified with the experimental data.

The problem of limited resistance of the composite material to surface pressures can be solved by using hybrid (mechanical–adhesive) joints, in which the adhesive layer, which is most stressed at the ends of the joint overlaps (area of mounting holes), significantly reduces the pressure of the fastener mandrel on the surface of the hole in the composite material, or, as it seems, by effectively using the friction phenomenon occurring between the joined elements in the area of mounting holes [24].

Based on the literature data, it is known that in mechanical joints, in transferring loads between connected elements, the phenomenon of friction is also considered [25,26]. In metal parts subjected to cyclic loads, the values of friction forces in the vicinity of mounting holes increase, which is a result of, among other things, the wear of mating surfaces of joined elements and the amount of wear products occurring.

In composite parts joined by mechanical fasteners, the load is also partly transferred by frictional forces [27,28,29] and the data presented in publications also indicate a regularity related to the increase in friction coefficient in the fatigue tests of joints where composite materials and mechanical fasteners were used [30]. In addition, some authors [28] suggest that the effect of increasing the coefficient of friction in cyclically loaded joints may compensate for the loss of assembly clamping, which is the result of the viscoelastic properties of the composite material and the change in the geometric dimensions of the joined components in the mechanical fastener assembly area.

The positive effect of frictional forces can be compounded by increasing the reaction field of the mounting pressures. Hence, numerous publications present the properties of joints using composite materials with mechanical fasteners and washers [31]. The effectiveness of the effect of mechanical washers on joint properties depends, among other things, on the geometry of the washers, which is directly related to the magnitude of the field of action of the assembly pressures [29,31].

The thesis of this paper is that in the case of the mechanical joining of composite materials, clamping forces, which are also involved in load transfer, will have a positive effect on reducing the surface pressures occurring between the fastener shank and the composite, and thus the fatigue life of the joints will be higher. Hence, the use of mechanical fasteners that induce a higher clamping force should prevent the degradation of the composite material in the area of the mounting holes and thus have a positive effect on the fatigue life of the joints [32,33].

Hence, the purpose of this study was to determine the impact of mechanical fastener types (effect of clamping forces) on the load capacity, fatigue life, and the mechanism of failure of mechanical joints of CFRP composite parts. Furthermore, it was decided to evaluate to what extent the hybridization of such joints, consisting of supplementing them with an adhesive joint, affects their static strength and the mechanism of failure of such joints loaded with fatigue. A key objective of the study was to determine the degradation mechanism of mechanical and hybrid joints of fatigue-loaded composite components, using computed tomography technology, which is one of the more effective methods for observing fatigue failure in composite materials [34,35]. Finally, in order to check how a partially cracked adhesive joint affects the load on the fasteners, numerical calculations were carried out. The innovation and research significance of this work lies in the possibility of the direct use of its results in engineering practice in the field of mechanical and hybrid connections using above-mentioned types of fasteners used in aviation. An important element of the work is to demonstrate the significant residual load capacity of partially damaged hybrid connections.

## 2. Research Object

**Figure 1:**
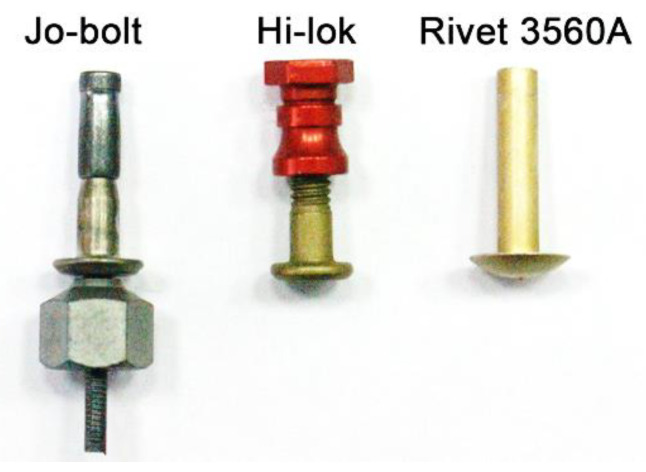
Fasteners used to prepare joints.

Parts cut from the composite material using the WaterJet technique were used in the experimental study. The composite material of the CFRP (carbon-fiber-reinforced polymer) type was made using autoclave technology from seven layers of prepreg type DF285 arranged according to the scheme [45°, 0°, 45°, 45°, 0°, 45°, 0°]. The composite curing conditions were as follows: heating to 120 °C with a 2 °C/min gradient, annealing at 120 °C for 90 min and cooling to 40 °C with a 2 °C/min gradient. Single-lap joints with an overlap length of 50 mm were prepared from cut pieces with dimensions of 100 mm length, 25 mm width, and 2.2 mm thickness. The following mechanical fasteners were used to join the elements: 3560A aluminum alloy rivets (diameter 4 mm), Hi-lok HL 1012 series (diameter 4.12 mm)-fasteners made of a titanium alloy with an aluminum collar, and one-sided Jo-Bolt fasteners (titanium-steel with diameter 4.12 mm)—Figure 1 [36,37].

**Figure 2:**
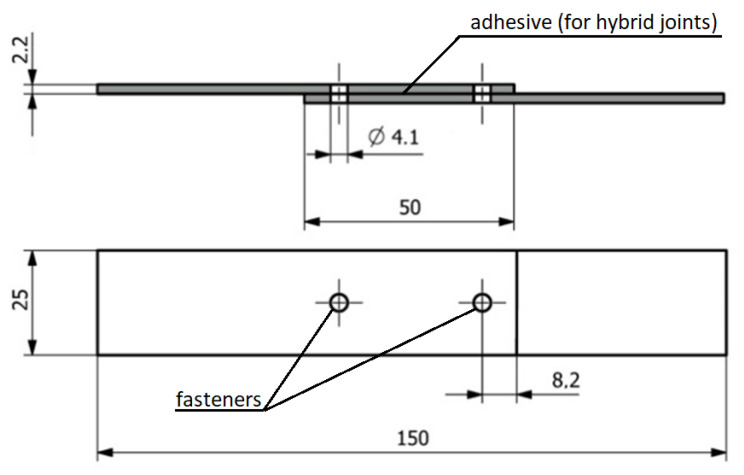
Connection diagram of composite materials in an experimental study.

The mounting holes in the 4.1 mm diameter specimens were made halfway across the width of the components to be joined and at a distance from the edge of the tabs equal to two diameters of the mounting fasteners. The connection diagram used in the experimental studies is presented in Figure 2.

**Figure 3:**
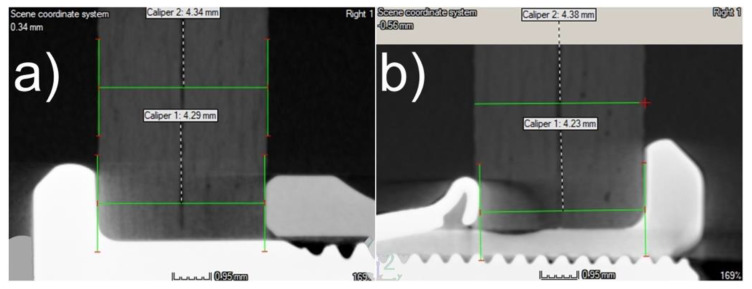
Illustrative tomographs of joints in the vicinity of mounting holes: (**a**) using Hi-lok fasteners; (**b**) using Jo-Bolt fasteners.

The fasteners used in this study differed not only in terms of the materials they were made of, but also in terms of the pressure forces they exerted on the joined parts near the mounting holes. A measurement of the thickness of the combined elements in the vicinity of the holes performed with the use of a CT scanner indicated that the highest pressures occurred after the assembly of the elements with Jo-Bolt fasteners. The measurements showed a reduction in specimen thickness at the fastener locations, averaging 1.6% in thickness for Hi-lok fasteners and averaging 3.1% for Jo-Bolt fasteners—Figure 3.

With deformations ε = 0.016, the pressure caused by the Hi-lok fastener was of the following order:

σ=ε×E=0.016×7000=112 MPa

where: *σ*—compress stress, ε—deformations, and *E* = 7000 MPa—the value of the longitudinal modulus of elasticity of the composite in the direction perpendicular to the fabric layers. However, at deformations ε = 0.031, the pressure caused by the Jo-Bolt fastener was:

σ=ε×E=0.031×7000=217 MPa

Knowing the surfaces of the fastener elements pressing directly on the composite, it is possible to estimate the pressure forces caused by the fasteners:

$$F_{Hi-Lok}=\sigma \times A\approx 112\times 30\approx 3360\;N$$

$$F_{Jo-Bolt}=\sigma \times A\approx 217\times 18\approx 3906\;N$$

where: *σ*—compress stress and *A*—surface area under the fastener head pressing directly on the composite.

Apart from the aforementioned mechanical fasteners, an Epidian 57/Z1 adhesive (Ciech Sarzyna S.A.) mixed with a Z1 hardener in a mass ratio of 10:1 was used to make hybrid (mechanical-adhesive) joints. The mechanical fasteners in the hybrid joints were installed identically to the mechanical joints, i.e., near the overlap end of the adhesive joint, which meant that they were installed in the zone where the adhesive joint was most stressed. The adhesive was cured in two stages, as recommended by the manufacturer, i.e., 24 h at room temperature and 8 h at 80 °C. The curing process of the adhesive bond took place after the installation of the mechanical fasteners. In order to maintain the thickness of the adhesive bond at 0.1 mm, spacer threads were inserted into the joint. The surfaces of the CFRP elements were prepared by washing with acetone, sanding with nonwoven abrasives, and washing again with acetone.

## 3. Research Methodology

In the first phase of experimental testing, the static capacity of the mechanical and hybrid joints was determined. The value of the load capacity of joints was used, inter alia, to define loads during fatigue (durability) tests. An MTS 809 Axial/Torsional testing machine was used for both static and fatigue tests. The bearing capacity of mechanical and hybrid joints was evaluated using three joint specimens. Static tests were performed at a rate of 2mm/min. Durability tests were realized within the variable load range with a frequency of 20 Hz. After a specified number of fatigue cycles, the connections were tested via computer tomography to assess the degree of failure of the joints.

## 4. Results of Static Tests

**Figure 4:**
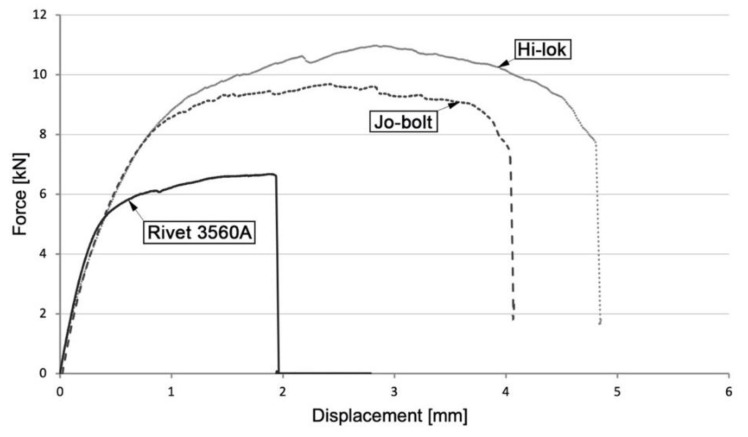
Tensile curve of mechanical joints.

The load capacity of mechanical joints determined in three tests for the variant with 3560 A aluminum alloy rivets was equal to: 6.68 kN, 6.83 kN, and 6.68 kN (mean value-6.73 kN); for the variant with Jo-Bolt fasteners: 9.30 kN, 9.69 kN, and 9.78 kN (mean value-9.59 kN); and for the variant with Hi-lok fasteners: 10.98 kN, 10.10 kN, and 10.25 kN (mean value-10.44 kN). Figure 4 presents an example of the tensile curves for mechanical joints.

**Figure 5:**
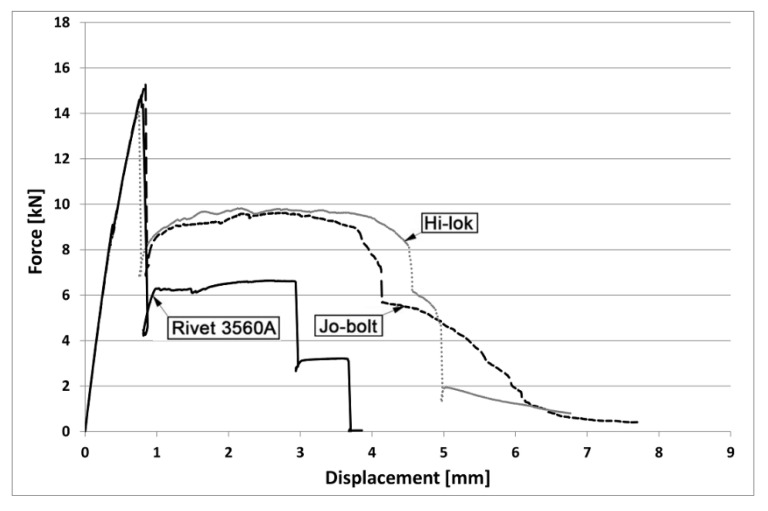
Tensile curve of hybrid joints.

The load carrying capacities of the hybrid joints with rivets were 14.80 kN, 14.30 kN, and 14.50 kN (mean value 14.53 kN). With Hi-lok fasteners, they were 14.07 kN, 14.20 kN, and 14.40 kN (mean value-14.22 kN), and with Jo-Bolt fasteners, 15.31 kN, 15.05 kN, and 15.40 kN (mean value-15.25 kN). Figure 5 shows the tensile curves of hybrid joints.

**Figure 6:**
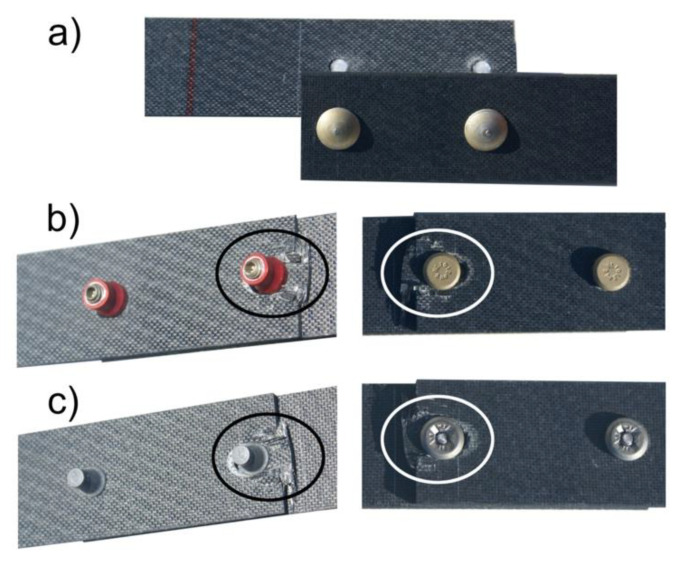
Forms of destruction of mechanical joints: (**a**) with rivets, (**b**) with Hi-lok fasteners, (**c**) with Jo-Bolt fasteners.

The load bearing capacity of the joints where Jo-Bolt and Hi-lok fasteners were used was significantly higher than the joints where rivets were used. Depending on the fastener used, various forms of joint destruction were also observed. The destruction of joints with 3560 A rivets occurred due to rivet shear, while joints with Hi-lok and Jo-Bolt fasteners occurred due to surface pressure and shear of the composite material—Figure 6.

The shear strength of 3560 A rivets was:

$$R_{t}=\frac{F}{2\pi \frac{d^{2}}{4}}=\frac{2\times 6730}{\pi \times 4^{2}}=268\;\mathrm{MPa}$$

In turn, surface pressures for 3560 A rivets were:

(1) $$p=\frac{F}{2\times g\times d}=\frac{6730}{2\times 2.2\times 4}=382\;\mathrm{MPa}$$

where: *d*—rivet diameter (forged rivet) and *g*—thickness of the composite plate.

Above calculated surface pressure did not cause visible damage to the surface of the holes in composite elements, so they can be considered acceptable. The composite failure pressures using Jo-Bolt fasteners were:

(2) $$p=\frac{F}{2\times g\times d}=\frac{9590}{2\times 2.2\times 4.1}=532\;\mathrm{MPa}$$

Similarly, the composite failure pressures when using Hi-lok fasteners were:

(3) $$p=\frac{F}{2\times g\times d}=\frac{10,440}{2\times 2.2\times 4.1}=579\;\mathrm{MPa}$$

The destruction of hybrid joints was carried out in two stages—first, the adhesive joint was destroyed under a similar load for all tested joints, and then the mechanical joints were destroyed under loads similar to those at which the mechanical joints were destroyed without the use of an adhesive layer. The use of hybridization increased the load capacity of riveted joints by 116%, Jo-Bolt by 16%, and Hi-lok by 14%.

## 5. Fatigue Tests

### 5.1. Test Conditions

In the fatigue tests for mechanical joints, the maximum load per cycle was assumed to be about 80% of the load carrying capacity of the fasteners, which meant a value of 5.5 kN for riveted joints, and a value of 8 kN for Hi-lok and Jo-bolt fasteners. The high maximum value of load per cycle was due to the fact that CFRP composite materials have an extremely long fatigue life.

In the hybrid joints with Hi-lok and Jo-Bolt joints, the maximum load per cycle was reduced to a value of about 70% of the joint load capacity, which, with the much higher load capacity of the hybrid joints, still meant a maximum load per cycle of 10 kN (2 kN higher than the mechanical joints). For fatigue tests of hybrid joints where rivets were applied, the cycle load values were identical to those of mechanical joints with rivets. This meant, inter alia, that the maximum load per cycle was equal to about 38% of the load capacity of the hybrid joint, due to the much higher load capacity of hybrid joints compared to mechanical joints. In the case of riveted hybrid joints, the principle of assuming maximum cycle loads of approximately 70% was abandoned, because in mechanical joints with rivets, it was the mechanical fasteners (rivets) that suffered fatigue failure. Thus, it was expected that in the case of hybrid joints with rivets, stressing them with a load of 70% could lead to a rapid failure of the joints (through rivet destruction). Table 1 shows the values of loads used in fatigue tests.

Tomographs of the joints subjected to fatigue testing were analyzed in the area of the mounting holes of the joints, according to the diagram presented in Table 2 and Table 3.

### 5.2. Fatigue Test Results

As was mentioned before, tomographs of the joints subjected to fatigue testing were analyzed in the area of the mounting holes. Already, a preliminary analysis of the tomographs indicated that the mechanical joints had a problem of joint destruction in the area of the mounting holes due to surface pressures. This is not apparent in the joints where rivets were used, because the fatigue tests on these types of joints had much lower loads than in the case when the joints with Hi-lok or Jo-Bolt fasteners were tested, and the rivets were sheared. In the case of mechanical joints with Hi-lok and Jo-Bolt fasteners, the loads were higher and consequently surface pressure issues arose. Among other things, ovalization of the mounting holes and angular displacement of the fasteners themselves (their rotation) were observed.

**Figure 7:**
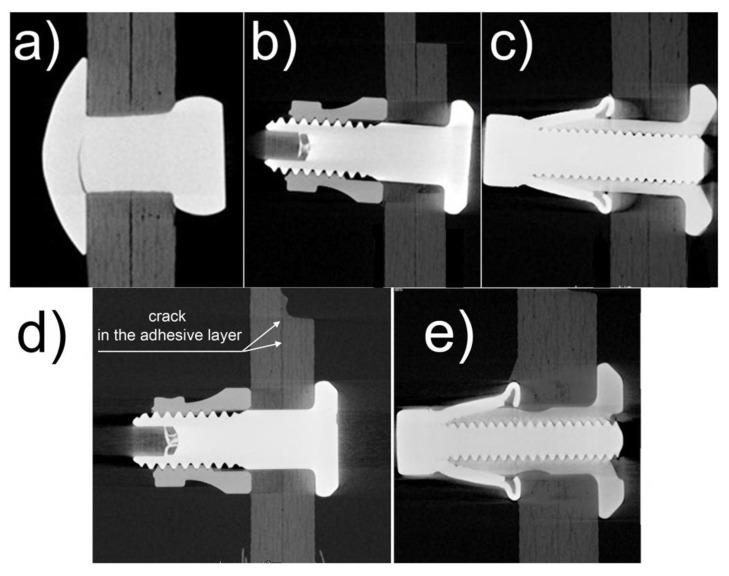
Tomographs of mechanical joints: (**a**) with rivets, (**b**) with Hi-lok fasteners, (**c**) with Jo-Bolt fasteners and hybrid joints: (**d**) with Hi-lok fasteners, (**e**) with Jo-Bolt fasteners.

There was no fastener rotation problem in the hybrid joints, but there were problems with fatigue cracking of the adhesive bond. Illustrative tomographs of mechanical and hybrid joints are presented in Figure 7.

### 5.3. Fatigue Test Results for Mechanical Joints–Rivets 3560A

**Figure 8:**
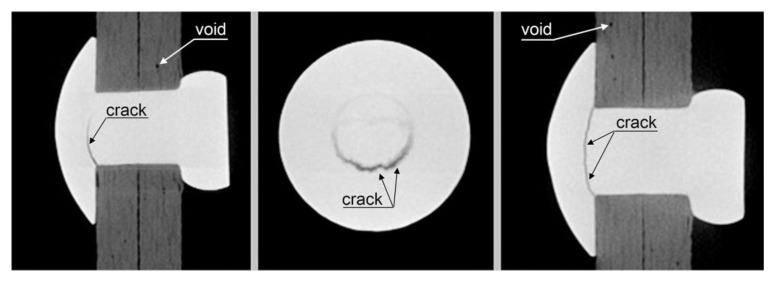
Tomographs of riveted mechanical joints following the fatigue tests (1.5 m cycles).

The riveted mechanical joint samples failed due to fatigue destruction of the rivets. The sample labeled NZ2 was destroyed after 1,831,415 cycles, while the sample labeled NZ1 was subjected to tomographic testing after 1.5 million cycles. The test detected fatigue cracks in the rivets in the absence of damage to the composite material. As you can see, the weakest element of the connection in this case were the rivets that failed at a fatigue load lower than that which causes damage to the composite due to the surface pressure in the rivet hole. The tomographs for sample NZ1 are presented in Figure 8.

### 5.4. Fatigue Test Results for Mechanical Joints–Hi-Lok Fasteners

**Figure 9:**
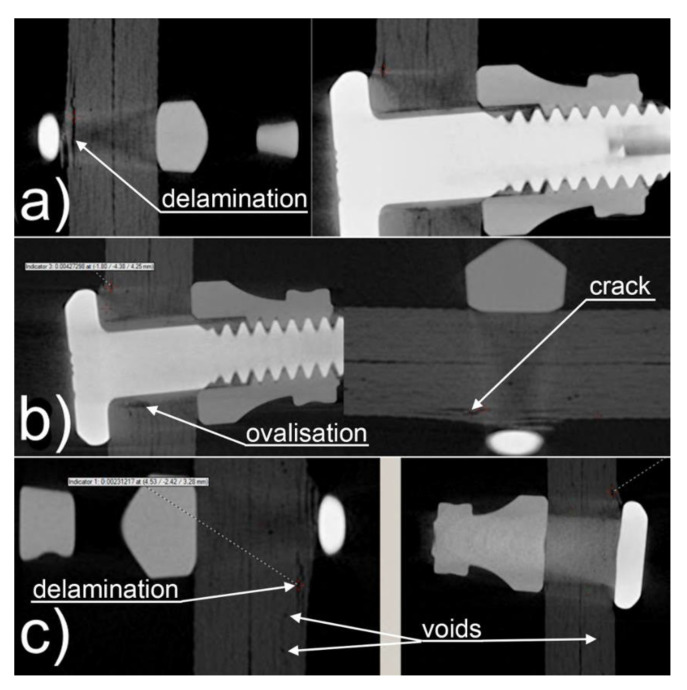
Examples of damage in mechanical joints with Hi-lok fasteners after fatigue tests (1.5 m cycles) in the form of: (**a**) delamination, (**b**) ovalization and cracks, (**c**) delamination and so-called voids.

Unlike with riveted mechanical joints, in the joints where Hi-lok fasteners were used, no damage was observed to the fasteners themselves, which had a much greater strength than ordinary rivets. However, there was damage to the joined parts near the mounting holes. The damage comprised primarily the ovalization of the holes and, in addition, cracks and local delamination. Some of the damage occurred directly underneath the fastener components and was invisible upon external visual assessment of the joint. Examples of failure characters for mechanical joints after fatigue testing (1.5 m cycles) with Hi-lok fasteners are presented in Figure 9.

**Figure 10:**
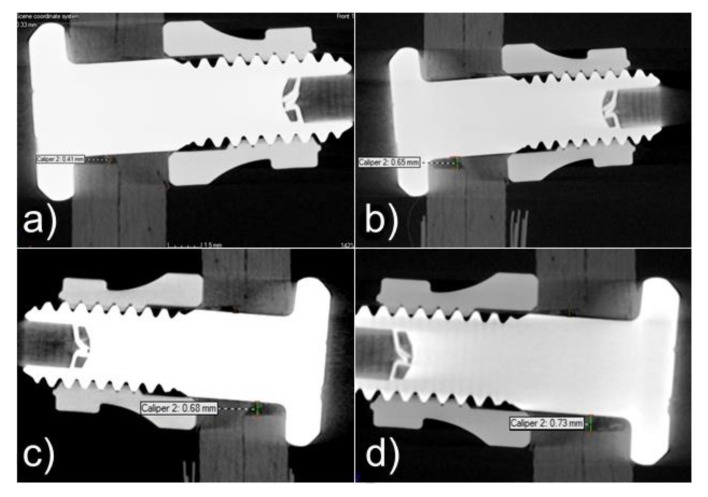
Illustrative changes in ovalization of mounting holes in mechanical joints with Hi-lok fasteners after fatigue tests: (**a**) after 1.5 m cycles, (**b**) after 4.5 m cycles, (**c**) after 7.5 m cycles, (**d**) after 12 m cycles.

Computed tomographic examination of joints after 1.5 m, 4.5 m, 7.5 m, and 12 m cycles showed increasing ovalization of the mounting holes with the number of cycles, which was a consequence of surface pressures appearing at the border between the lateral surface of the hole and the mechanical fastener pin. The increase in hole ovalization also caused the increasing angular deflection (rotation) of the mechanical fastener in the mounting hole. The observed changes are presented in Figure 9, whereas Figure 10 shows the average values of hole ovalization change for mechanical joints with Hi-lok fasteners and with Jo-Bolt fasteners. Larger hole ovalization in the case of Hi-lok fasteners compared to Jo-Bolt is due to the fact that Hi-lok fasteners have a smaller head diameter and the collar on one side is made of aluminum, while Jo-Bolt fasteners are a titanium and steel construction.

### 5.5. Fatigue Test Results for Mechanical Joints–Jo-Bolt Fasteners

**Figure 11:**
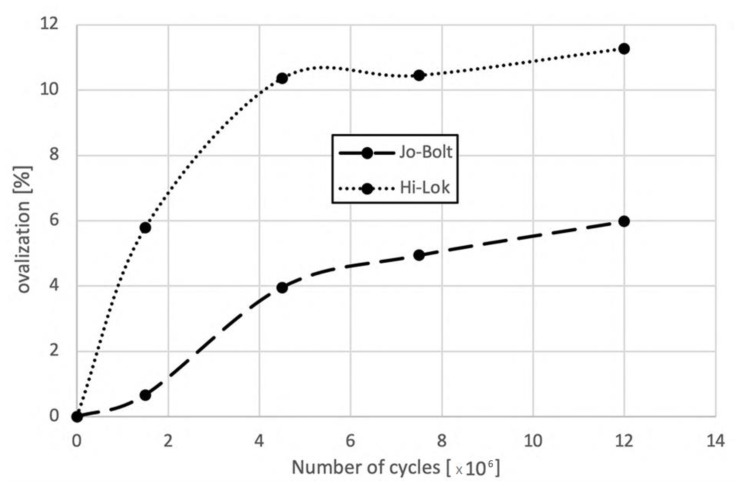
Increase in mounting hole ovalization in mechanical joints with Hi-lok and Jo-Bolt fasteners with increasing number of fatigue cycles.

**Figure 12:**
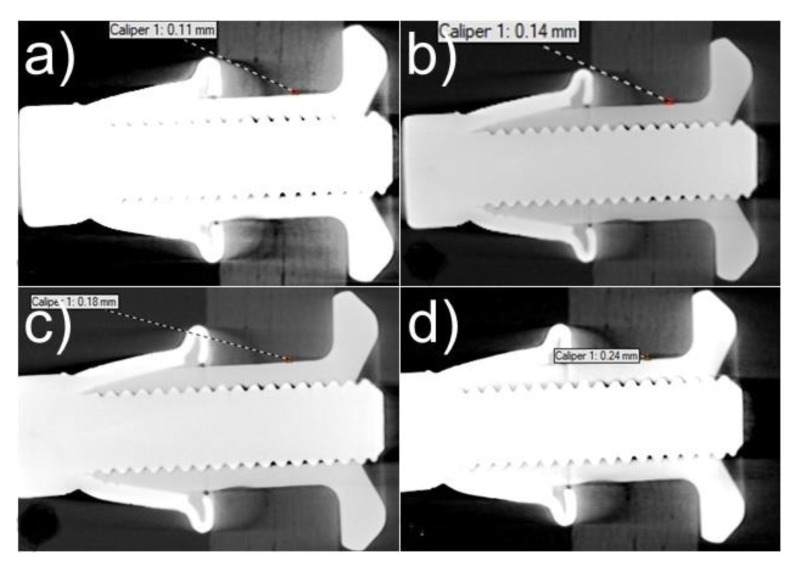
Illustrative tomographs of mechanical joints with Jo-Bolt type fasteners near mounting holes after fatigue tests: (**a**) after 1.5 m cycles, (**b**) after 4.5 m cycles, (**c**) after 7.5 m cycles, (**d**) after 12 m cycles.

In the case of mechanical joints with Jo-Bolt fasteners, the ovalization of the holes was about twice as low (cf. Figure 11) and, additionally, no damage in the CFRP material in the form of delamination or cracks was observed in the vicinity of the mounting holes (Figure 12).

To sum up, the failure characters for mechanical joints after fatigue testing are presented in Table 4.

### 5.6. Fatigue Test Results for Hybrid Joints–3560A Rivet Fasteners

**Figure 13:**
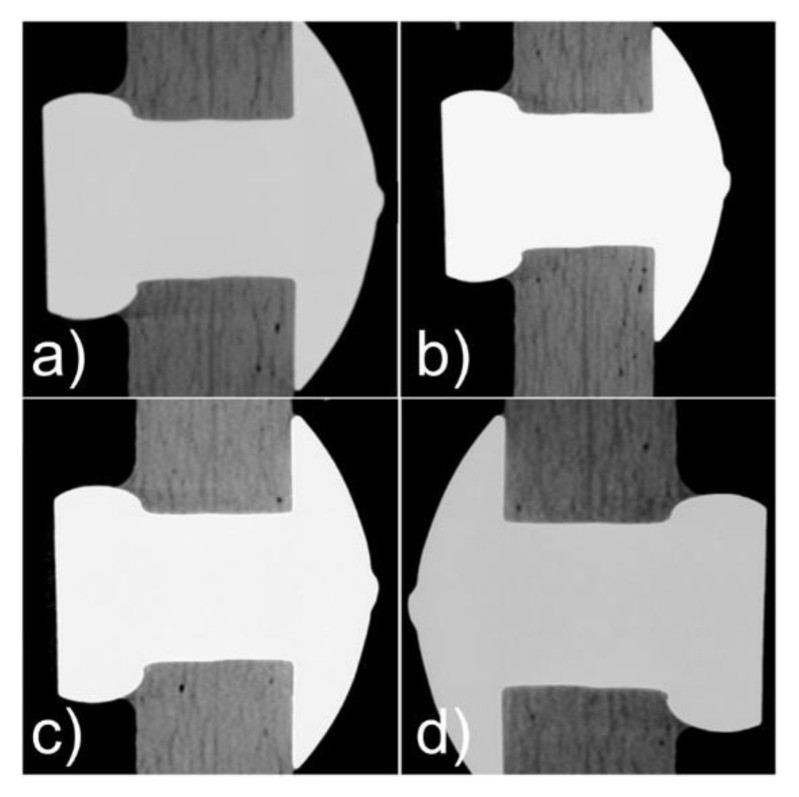
Illustrative tomographs of hybrid joints with rivets near mounting holes after fatigue tests: (**a**) after 1.5 m cycles, (**b**) after 4.5 m cycles, (**c**) after 7.5 m cycles, (**d**) after 12 m cycles.

In the hybrid joints prepared using 3560A rivets and Epidian 57/Z1 adhesive, no complete destruction of the joint was observed, as was the case in the mechanical joint. After 3 million cycles, there was partial cracking of the outer edges of the adhesive joint, but even after 12 million cycles, there was no sign of further deterioration of the joints. Note, however, that the maximum cycle load was equal to approximately 38% of the hybrid connection capacity in this study. The partial cracking of the adhesive joint at the outer edges of the joint is due to the secondary bending action in the lap joint. Figure 13 presents example tomographs of hybrid joints in the area of mounting holes after fatigue tests in the range of 1.5 to 12 m cycles.

### 5.7. Fatigue Test Results for Hybrid Joint-Hi-Lok and Jo-Bolt Fasteners

**Figure 14:**
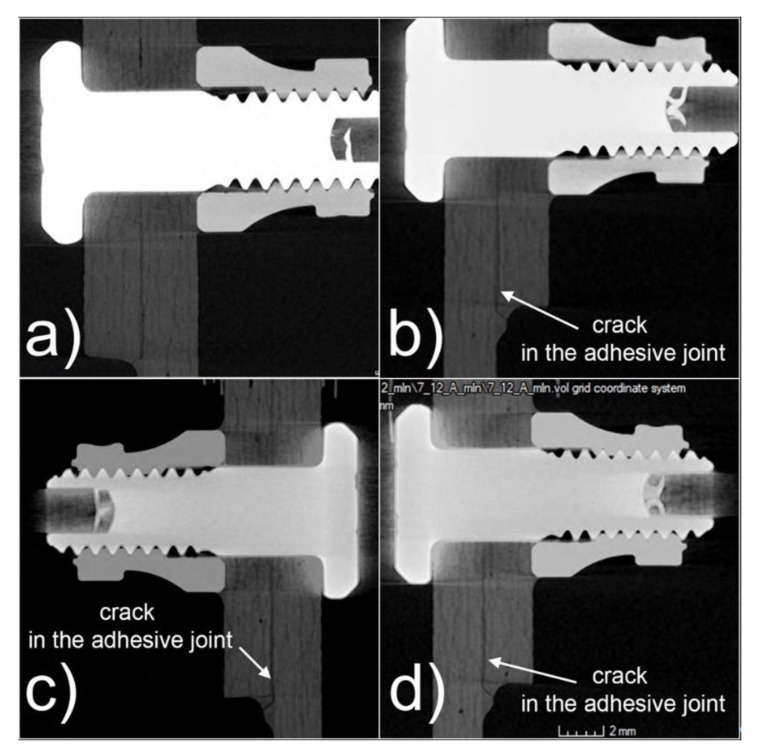
Illustrative tomographs of hybrid joints with rivets near mounting holes after fatigue tests: (**a**) after 1.5 m cycles, (**b**) after 4.5 m cycles, (**c**) after 7.5 m cycles, (**d**) after 12 m cycles.

Specimens using the hybrid Hi-lok/adhesive and Jo-Bolt adhesive, after being loaded with 12 m fatigue cycles, showed no ovalization or deviation of the joint. The fasteners’ structure was not affected either. The only visible defect in the samples was cracking of the adhesive bond at the ends of the bonded parts. Partial cracking of the adhesive joint at the outer edges of the joint is due to the secondary bending action in the lap joint. Tomographs taken after successive fatigue cycles of the hybrid Hi-lok/adhesive joint can be seen in Figure 14.

## 6. Discussion of Experimental Research Results

In the tested lap joints, the fasteners and adhesive joints were subjected to shear stress and, due to the effect of secondary bending moments, the heads of mechanical fasteners and the adhesive joints were torn off. In the static tests of riveted joints, the rivets were sheared under their pressure on the walls of the holes, which did not cause any visible damage to these holes. Based on the test results, it can be concluded that the pressure of 380 MPa can be considered acceptable for the tested composite. In the fatigue tests, in which the value of the maximum loads was at the level of 86% of the destructive loads, the fatigue tearing off of the rivet heads occurred. In these tests, in which the level of maximum nominal stresses in the cross-section with a hole was of the order of 105 MPa, no visible damage to the composites was found.

The analysis of tomograms of connections with Jo-Bolt and Hi-lok fasteners shows that these fasteners exert high pressures of heads and nuts on the connected elements. Hence, there are friction forces between these elements that are involved in the transfer of loads. Due to the fact that the shanks of these connectors are made of high-strength materials, they do not deteriorate, and at pressures of 530 MPa, they destroy the joined composite elements.

In fatigue tests of mechanical joints with Jo-Bolt and Hi-lok connectors, the maximum nominal stresses in the cross-section with the hole were of the order of 152 MPa. The applied load was probably greater than the friction forces, which caused the shanks of the fasteners to cyclically press on the holes, causing their ovalization, and the destruction of the edges of the holes. This resulted in the shifting of the connected elements relative to each other and the skewing of the fasteners.

The applied hybridization of mechanical joints, consisting of adding adhesive joints to them, resulted in a significant increase in their temporary strength, the greatest in the case of riveted joints. The two-stage nature of the destruction of these connections was characteristic and significantly increases the safety of aircraft structures and facilitates the process of supervising their technical condition. The strength of all tested hybrid joints was similar (only 5% higher for Jo-Bolt) and resulted from the strength of the adhesive joint. The mechanical fasteners used significantly strengthened the adhesive connection.

In the fatigue tests of the rivet–adhesive hybrid connection, the same fatigue load cycle was used as for the riveted connection. After 3 million cycles, there was partial cracking of the outer edges of adhesive joint, but even after 12 million cycles, there was no sign of further deterioration of the joints.

Hybrid joints with Hi-lok and Jo-Bolt fasteners were loaded with a fatigue cycle, in which the maximum loads were 25% higher than those used in the mechanical connection tests. After 3 million cycles, cracks were found in the adhesive joints; however, even after 12 million cycles, no further damage occurred. The cracks in the joints occurred in the sections of the connection edge-holes. Maintaining the continuity of the adhesive joint between the holes for mechanical fasteners prevented further fatigue damage of the joints.

## 7. Numerical Calculations

In the fatigue tests of the rivet–adhesive hybrid connection, there was partial cracking of the outer edges of the adhesive joint, but after millions of cycles, there was no sign of further deterioration of the joints. Therefore, authors checked whether a partial crack in the adhesive joint causes significant loading of the mechanical fasteners. The comparative numerical calculations of hybrid connections with Hi-lok fasteners with an undamaged joint and 6.7 mm long cracks on the connection edges were carried out. Numerical calculations were made in the CAE Ansys Workbench 2021 R2 program using the Static Structural module. The joint was loaded with the maximum force used in the fatigue tests (10 kN) and the pressures from the heads of the fasteners were modeled by declaring the initial load using the “Bolt Pretension” function with a force value of 3500 N.

The fasteners were given the linear elastic properties of titanium, the adhesive bilinear properties of Epidian 57, and the composite elements of the orthotropic properties of a carbon–epoxy composite (Table 5 and Table 6).

**Figure 15:**
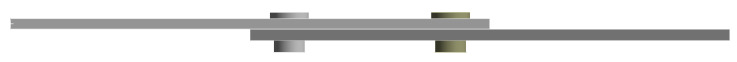
The lap joint model in the Geometry Ansys module.

The lap joint models (Figure 15) consisted of two plates with dimensions of 25 × 100 × 2.2 mm (width × length × thickness), connected with two fasteners with a diameter of 4.1 mm, with the first model having an adhesive joint measuring 50 × 25 × 0.12 mm (undamaged), while in the second model, it measured 36.6 × 25 × 0.12 mm (damaged).

**Figure 16:**
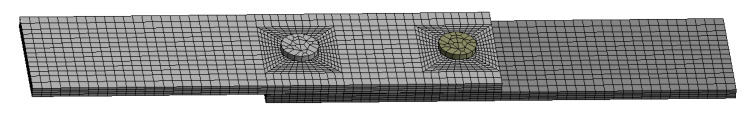
Discretized numerical model of the sample.

The presence of frictional contact with a coefficient of friction 0.1 is defined between the fasteners and the adhesive joints and the connected plates. Bonded contact has been defined between the adhesive joint and the connected plates. For most of the model, the side dimension of the mesh elements is 2 mm, while near the holes, the mesh is concentrated (Figure 16). After discretizing the geometry, a model consisting of 12,062 Hex20 elements (non-linear hexagonal with 20 nodes) and 63,504 nodes was obtained.

**Figure 17:**
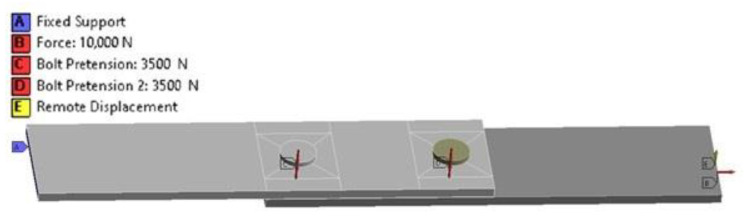
Boundary conditions of the model (A–restraint, B–load, C and D preload in fasteners, E–displacement/restraint).

One end of the sample was restrained using the function Fixed Support (all six degrees of freedom were taken away$\;\left(D_{X},D_{Y},D_{Z}=0;\;R_{X},R_{Y},\;R_{Z}=0\right)$. The other end of the sample was restrained using the function Remote Displacement in the direction of *X* axis and was deprived of the ability to rotate in all directions. It was also given a displacement in the direction *Z* 2,2 mm, which corresponds to the thickness of one connected plate $\left(D_{X}=0,\;D_{Z}=2.2\;\mathrm{mm};\;R_{X\;},R_{Y},\;R_{Z}=0\right)$. The rivets were preloaded by force 3500 N with the use of the Ansys-Bolt Pretension function. This corresponds to the force with which the fasteners clamp the connected plates. The sample tensile load of 10,000 N was assigned to the right end of the sample (Figure 17).

**Figure 18:**
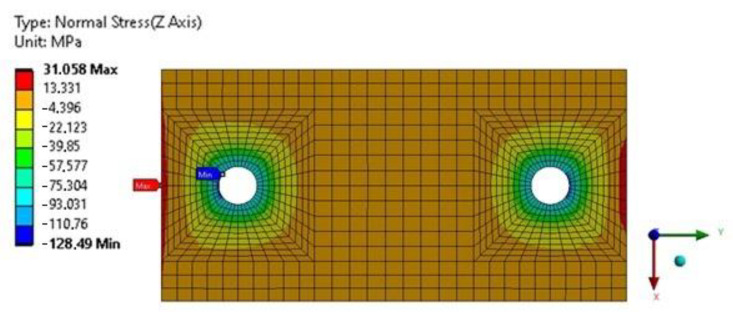
Map of normal stresses in the Z direction in a 50 mm long adhesive joint.

**Figure 19:**
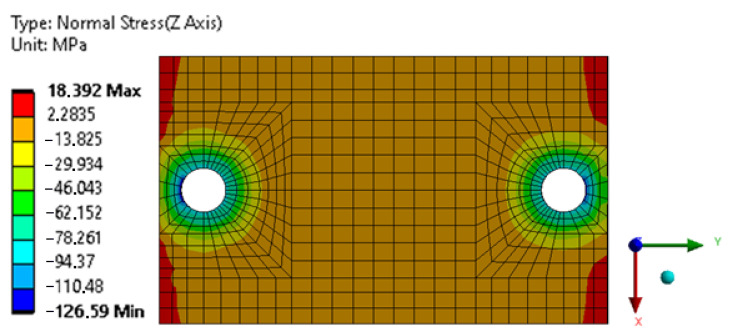
Map of normal stresses in the Z direction in the adhesive joint with a length of 33.6 mm.

The values of normal stresses perpendicular to the surface of the adhesive joint causing its peeling, were calculated and compared for: adhesive joints that were 50 mm long (undamaged) and 33.6 mm long (damaged)—Figure 18 and Figure 19.

The calculations show a significant decrease (about 40%) of stresses causing peeling in the shorter adhesive joint, which shows that the process of joint destruction can be inhibited.

**Figure 20:**
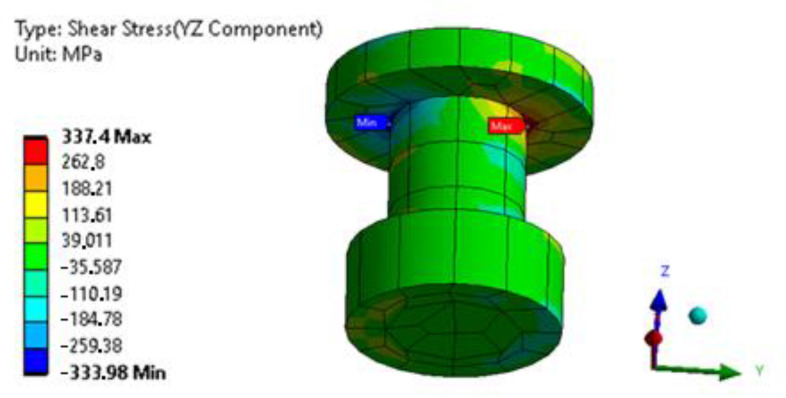
Map of shear stresses in the fastener of the undamaged connection.

**Figure 21:**
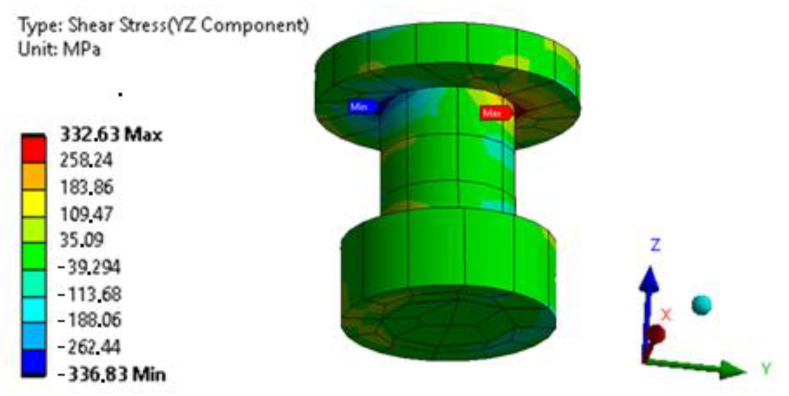
Map of shear stresses in the fastener for modeled cracks on the edge of the adhesive joint.

Figure 20 and Figure 21 compare the shear stresses in the mechanical fasteners of the undamaged connection and the connection with modeled cracks in the edges of the adhesive joints.

The calculations show that partial damage to the adhesive joint of the hybrid joint does not increase the load on the rivets.

## 8. Conclusions

Hi-lok and Jo-Bolt fasteners, due to the materials from which they are made and relatively high clamping forces that compress the joined elements, allow for a greater strength of connections of the composite elements compared to ordinary solid rivets made of aluminum alloys.

The tests carried out show that the carbon composite used in the tests is characterized by a resistance to pressure caused by mechanical fasteners at the level of at least 380 MPa under static loads.

Fatigue damage of the mechanical connections of composite elements may consist either of the destruction of low-strength fasteners or of the destruction of the composite by delamination, chipping of the hole edges, their ovalization, and skewing of the fasteners.

The hybridization of mechanical connections, by adding adhesive connections to them, increases their static strength to a greater extent than connections with lower strength.

The greatest benefits of hybridization of joints relate to their fatigue life. The important finding of the paper is that the partial destruction of the adhesive joint on the edge caused by its peeling does not cause the entire load to be transferred by the mechanical fasteners. If the undamaged adhesive joint between the fasteners is of the appropriate length, it effectively prevents the connected elements from moving relative to each other, transfers some of the load, and reduces the pressure of the mechanical fasteners on the holes, which effectively increases the fatigue life of the connections. Two-stage destruction of the hybrid connection significantly increases the safety of aircraft structures and facilitates the process of supervising their technical condition.

## Figures and Tables

**Table 1:** Load values of joints in fatigue tests.

Fastener Type	Load Capacity $F_{max}$ [kN]	Load During the Fatigue Cycle $F_{min}÷F_{max}$ [kN]	Average Load of the Cycle [kN]	Cycle Asymmetry Coefficient	$\frac{F_{zmax}}{F_{max}}$
Rivets 3560A	6.37	3.5 ÷ 5.5	4.5	0.64	0.863
Hi-lok HL 1012	10.44	5 ÷ 8	6.5	0.63	0.766
Jo-Bolt	9.59	5 ÷ 8	6.5	0.63	0.834
Rivets 3560A/Epidian 57	14.53	3.5 ÷ 5.5	4.5	0.64	0.379
Hi-lok HL 1012/Epidian 57	14.22	7 ÷ 10	8.5	0.7	0.703
Jo-Bolt/Epidian 57	15.23	7 ÷ 10	8.5	0.7	0.657

**Table 2:** Diagram of tomographic testing of mechanical joints.

Fastener Type	Sample No.	Load Range within the Cycle [kN]	No. of Cycles /T/- Tomograph
Rivets made of aluminum alloy 3560A	NZ1	3.5 ÷ 5.5	1.5 m/T/
	NZ2	3.5 ÷ 5.5	1.831.415
Hi-lok	HZ1	5 ÷ 8	0/T/
			1.5 m/T/
			4.5 M/T/
			7.5 M/T/
			12 M/T/
	HZ2	5 ÷ 8	0/T/
			1.5 m/T/
			4.5 M/T/
			7.5 M/T/
			12 M/T/
	HZ3	5 ÷ 8	0/T/
			4.5 M/T/
			9 M/T/
			12 M/T/
Jo-Bolt	JZ1	5 ÷ 8	0/T/
			1.5 m/T/
	JZ2	5 ÷ 8	0/T/
			1.5 m/T/
			4.5 M/T/
			7.5 M/T/
			12 M/T/
	JZ3	5 ÷ 8	0/T/
			4.5 M/T/
			9 M/T/
			12 M/T/

**Table 3:** Diagram showing how to perform tomographic testing of hybrid joints.

Fastener Type	Sample No.	Cycle Fatigue Loads [kN]	Cycle and Cycle Intervals /T/- Tomograph
Rivets made of aluminum alloy 3560A	HNZ1	3.5–5.5	0/T/
			3 M/T/
			6 M/T/
			9 M/T/
			12 M/T/
Hi-lok	HHZ1	7–10	0/T/
			3 M/T/
			6 M/T/
			9 M/T/
			12 M/T/
Jo-Bolt	HJZ1	7–10	0/T/
			3 M/T/
			6 M/T/
			9 M/T/
			12 M/T/

**Table 4:** Failure characters for mechanical joints after fatigue testing.

Fastener Type	Sample No.	Load Range within the Cycle [kN]	No. of Cycles /T/- Tomograph	Status of the Sample						
				Delamination	Cracks in the Material	Ovalization	Joint Damage	No Breaking	Sample Destruction	End of Testing
Rivets made of aluminum alloy 3560A	NZ1	3.5–5.5	1.5 m/T/				X			X
	NZ2	3.5–5.5	1.831.415				X		X	X
Hi-lok	HZ1	5–8	0/T/					X		
			1.5 m/T/	X	X	X				
			4.5 M/T/	X	X	X				
			7.5 M/T/	X	X	X				
			12 M/T/	X	X	X				X
	HZ2	5–8	0/T/					X		
			1.5 m/T/	X	X	X				
			4.5 M/T/	X	X	X				
			7.5 M/T/	X	X	X				
			12 M/T/	X	X	X				X
	HZ3	5–8	0/T/					X		
			4.5 M/T/	X	X	X				
			9 M/T/	X	X	X				
			12 M/T/	X	X	X				X
Jo-Bolt	JZ1	5–8	0/T/					X		
			1.5 m/T/					X		X
	JZ2	5–8	0/T/					X		
			1.5 m/T/			X				
			4.5 M/T/			X				
			7.5 M/T/			X				
			12 M/T/			X				X
	JZ3	5–8	0/T/					X		
			4.5 M/T/			X				
			9 M/T/			X				
			12 M/T/			X				X

**Table 5:** Material constants of mechanical fasteners and adhesives.

Type of Material	Young’s Modulus [GPa]	Poisson’s Ratio [-]	Kirchhoff Modulus [GPa]	Elastic Limit [GPa]	Strengthening Modulus [GPa]
Titanium Alloy	96	0.36	35.29	-	-
Epidian 57	2.083	0.35	0.771	0.05828	0.49057

**Table 6:** Material constants of orthotropic carbon laminate.

		Young’s Modulus [GPa]			Poisson’s Ratio [-]			Kirchhoff Modulus [GPa]		
Type of material	Direction	X	Y	Z	XY	YZ	XZ	XY	YZ	XZ
Composite laminate		42.41	42.41	7	0.04	0.3	0.3	2.36	1.94	1.94

## Data Availability

Not applicable.
